# Optimally timing primaquine treatment to reduce *Plasmodium falciparum *transmission in low endemicity Thai-Myanmar border populations

**DOI:** 10.1186/1475-2875-8-159

**Published:** 2009-07-15

**Authors:** Saranath Lawpoolsri, Eili Y Klein, Pratap Singhasivanon, Surapon Yimsamran, Nipon Thanyavanich, Wanchai Maneeboonyang, Laura L Hungerford, James H Maguire, David L Smith

**Affiliations:** 1 Department of Epidemiology and Preventive Medicine, University of Maryland School of Medicine, Baltimore, Maryland, USA; 2 Department of Tropical Hygiene, Faculty of Tropical Medicine, Mahidol University, Bangkok, Thailand; 3 Department of Ecology and Evolutionary Biology, Princeton University, Princeton, New Jersey, USA; 4 Department of Biology, University of Florida, Gainesville, FL, USA; 5 Emerging Pathogens Institute, University of Florida, Gainesville, FL, USA

## Abstract

**Background:**

Effective malaria control has successfully reduced the malaria burden in many countries, but to eliminate malaria, these countries will need to further improve their control efforts. Here, a malaria control programme was critically evaluated in a very low-endemicity Thai-Myanmar border population, where early detection and prompt treatment have substantially reduced, though not ended, *Plasmodium falciparum *transmission, in part due to carriage of late-maturing gametocytes that remain post-treatment. To counter this effect, the WHO recommends the use of a single oral dose of primaquine along with an effective blood schizonticide. However, while the effectiveness of primaquine as a gametocidal agent is widely documented, the mismatch between primaquine's short half-life, the long-delay for gametocyte maturation and the proper timing of primaquine administration have not been studied.

**Methods:**

Mathematical models were constructed to simulate 8-year surveillance data, between 1999 and 2006, of seven villages along the Thai-Myanmar border. A simple model was developed to consider primaquine pharmacokinetics and pharmacodynamics, gametocyte carriage, and infectivity.

**Results:**

In these populations, transmission intensity is very low, so the *P. falciparum *parasite rate is strongly linked to imported malaria and to the fraction of cases not treated. Given a 3.6-day half-life of gametocyte, the estimated duration of infectiousness would be reduced by 10 days for every 10-fold reduction in initial gametocyte densities. Infectiousness from mature gametocytes would last two to four weeks and sustain some transmission, depending on the initial parasite densities, but the residual mature gametocytes could be eliminated by primaquine. Because of the short half-life of primaquine (approximately eight hours), it was immediately obvious that with early administration (within three days after an acute attack), primaquine would not be present when mature gametocytes emerged eight days after the appearance of asexual blood-stage parasites. A model of optimal timing suggests that primaquine follow-up approximately eight days after a clinical episode could further reduce the duration of infectiousness from two to four weeks down to a few days. The prospects of malaria elimination would be substantially improved by changing the timing of primaquine administration and combining this with effective detection and management of imported malaria cases. The value of using primaquine to reduce residual gametocyte densities and to reduce malaria transmission was considered in the context of a malaria transmission model; the added benefit of the primaquine follow-up treatment would be relatively large only if a high fraction of patients (>95%) are initially treated with schizonticidal agents.

**Conclusion:**

Mathematical models have previously identified the long duration of *P. falciparum *asexual blood-stage infections as a critical point in maintaining malaria transmission, but infectiousness can persist for two to four weeks because of residual populations of mature gametocytes. Simulations from new models suggest that, in areas where a large fraction of malaria cases are treated, curing the asexual parasitaemia in a primary infection, and curing mature gametocyte infections with an eight-day follow-up treatment with primaquine have approximately the same proportional effects on reducing the infectious period. Changing the timing of primaquine administration would, in all likelihood, interrupt transmission in this area with very good health systems and with very low endemicity.

## Background

*Plasmodium falciparum *is endemic in 87 countries and approximately 2.4 billion persons are at risk [1,2]. As populations in South Asia and South East Asia regions have grown, so have the number of people at risk [3-5]. Now, approximately one billion people live in areas at very low risk of *P. falciparum *[2,4]. Recently, the WHO Global Malaria Programme has called for countries with low and moderate transmission areas to eliminate malaria transmission from their entire territory [6]. In many of these areas, malaria transmission is suppressed through a combination of insecticide-treated nets, indoor residual spraying, and prompt, effective treatment with anti-malarial drugs [1]. However, despite implementation of all the control measures, in some areas, such as the border region with Myanmar in Thailand, malaria transmission persists. This suggests a need to critically re-examine malaria epidemiology and the parasite life cycle to identify new control points.

One of the components of an elimination programme is to reduce transmission from malaria patients by making the infectious period as short as possible [6]. Prompt treatment of clinical cases is important, but in humans, mature gametocytes are the only infective stage of malaria parasite, and most drugs do not kill mature gametocytes [7]. In addition, in the *P. falciparum *life-cycle, unlike in other *Plasmodium spp*., the appearance of infective gametocytes is delayed with respect to the erythrocytic-schizogony cycle, resulting in a delayed appearance of mature *P. falciparum *gametocytes in the peripheral blood about 7–15 days after the initial acute attack [7,8]. While, an untreated infection lasts about six months, on average, this duration can be cut short to a few days after the incubation period [7]. Thus, in areas where asexual parasitaemia is cut short by effective treatment with anti-malarial drugs and gametocyte production is, therefore, limited, lingering gametocytes can maintain malaria transmission [9-11]. This issue is particularly important in areas with good access to medical clinics.

Although artemisinin derivatives have been shown to reduce gametocyte carriage by eliminating asexual parasites and immature gametocytes, only the 8-aminoquinolines, such as primaquine, are lethal to the mature gametocytes [7,12]. Primaquine, which has been shown to be effective against mature *P. falciparum *gametocytes is rapidly absorbed with peak plasma concentrations reached about two hours after administration [13,14]. However, it has a drug elimination half-life of approximately eight hours, so it only remains active against parasites for, at most, a few days [12,15]. The WHO recommends the use of a single oral dose of primaquine along with an effective blood schizonticide to reduce transmission, particularly in low endemic areas [12]. In areas where an early detection and treatment programme is highly effective, patients generally receive treatment one or two days after the acute attack, or approximately three to five days before gametocyte maturation [16]. While the effectiveness of primaquine as a gametocidal agent is widely documented, the mismatch between primaquine's short half-life, the long-delay for gametocyte maturation, and the proper timing of primaquine administration have not been studied.

Here, a mathematical model was constructed to describe *P. falciparum *transmission dynamics in seven hamlets along the Thai-Myanmar border, an area with low, seasonal transmission, and a highly effective health system. The transmission model was extended to investigate the timing of follow-up primaquine administration on the duration of gametocyte carriage and the implications for malaria transmission in the area. The models were developed with the goal of critically evaluating malaria transmission and tailoring control measures in areas with extremely good health systems, where elimination of malaria is feasible.

## Methods

### Study site

A malaria transmission model was developed to closely track the observed transmission intensity over an eight-year period of seven hamlets in the Tanaosri subdistrict, Suanphung district in Ratchaburi Province, a mountainous area along the Thai-Myanmar border. The study site covered an area of around 50 km^2 ^with approximately 3,500 inhabitants in about 500 households. Free diagnosis and treatment of malaria has been provided for people in the area since 1997 by the Rajanagarindra Tropical Disease International Center (RTIC), operated by Mahidol University, Thailand, the only malaria clinic in this area. Individuals commonly receive treatment for febrile malaria within two days of an acute attack. After deployment of the early detection and treatment programme, the peak incidence of clinical malaria has gradually decreased from approximately four cases per 100 persons in 1999 to about two cases per 100 persons from 2003 through 2005. A single dose of mefloquine had been used as the first-line drug for treatment of uncomplicated falciparum malaria until the year 2005; the standard regimen was then changed to a two-day artesunate-mefloquine combination therapy, as recommended by the Thai government, because of the increased mefloquine resistance in the area [17]. A single dose of 30 mg primaquine is given to all *P. falciparum *positive patients on the last day of the treatment course. In Thailand, the haemolysis after primaquine administration among glucose-6-phosphate dehydrogenase (G6PD) deficiency patients is relatively mild [18,19]. A test for G6PD deficiency is not required before a single dose primaquine administration.

Malaria transmission in this area is markedly seasonal with a peak of transmission during the rainy season (April to July), mainly due to the fluctuation of the mosquito population. However, malaria transmission does continue at a low level during the dry season. The *P. falciparum *parasite rate (*Pf*PR) in the area is very low, according to regular active surveillance surveys that have sampled more than half of the study population since 2003 (Table 1). Parasites were not detected in three surveys in 2005. Although some infections with low parasite densities might have gone undetected, the low *Pf*PR and yearlong continuous transmission suggest that asexual blood-stage infection is rare and thus gametocyte carriage in the absence of asexual parasites must play an important role in maintaining mosquito infection and the persistence of malaria transmission in the area.

**Table 1:** *Plasmodium falciparum *parasite rate (*Pf*PR) according to active surveillance surveys in the study area between 2003 and 2005.

Year	Census	Survey month	Number of blood samples	*# P. falciparum *positive	*Pf*PR
**Year 2003**	3059	June	1675	25	1.49
		August	1723	12	0.70
		October	1774	11	0.62
		November	1880	7	0.37
		December	1836	5	0.27
**Year 2004**	2906	February	1764	7	0.40
		May	1836	4	0.22
		June	1335	17	1.27
		September	1798	12	0.67
		November	1815	3	0.17
**Year 2005**	2990	January	1798	3	0.17
		March	1801	0	0.00
		May	1653	0	0.00
		July	1647	0	0.00

### Transmission model

**Figure 1:**
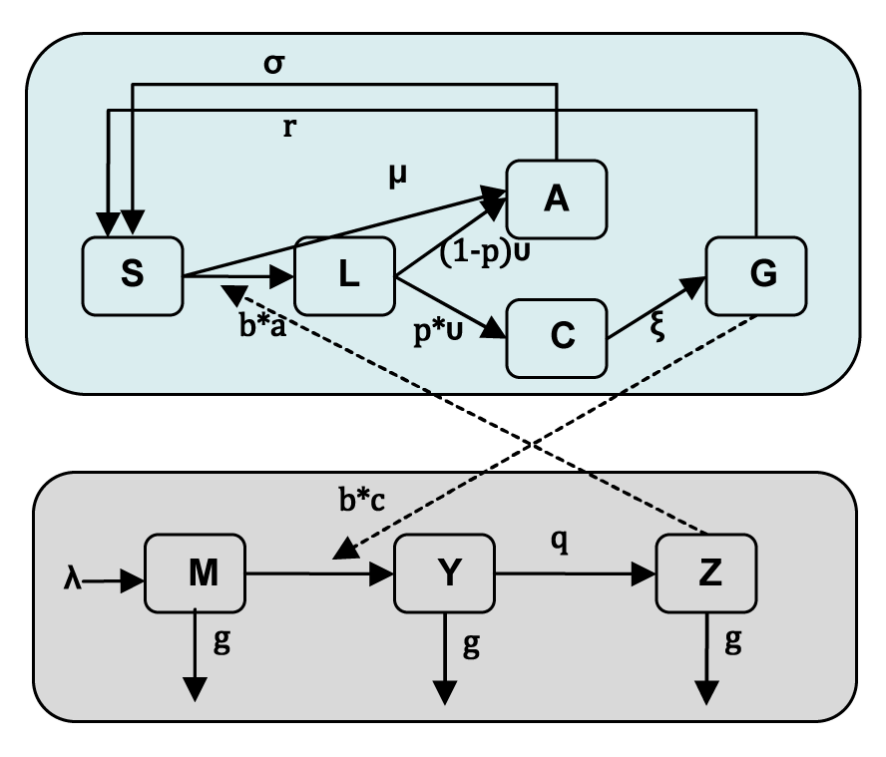
**Schematic illustration of the malaria transmission model**. The diagram shows the compartments of human population (Top) and mosquito population (Bottom) and transmission processes. For human population: S_h _= susceptible; L_h _= liver-stage only; A_h _= asymptomatic infection with gametocyte carriage; C_h _= clinical episode; and G_h _= gametocyte carriage only. For mosquito population: M = susceptible; Y = infected; and Z = infectious.

A deterministic model of the infection dynamics of human and mosquito populations in the study area was developed. In the models, the human population was divided into five compartments: state variables tracked the proportion that were susceptible (*S*_*h*_), liver-stage only (*L*_*h*_), asymptomatic infection with gametocyte carriage (*A*_*h*_), clinical episode (*C*_*h*_), and gametocyte carriage only (*G*_*h*_). In this low malaria transmission setting, malaria immunity and super-infection are rare, so an immune population was not included in the model. In the model for the mosquito population, seasonal mosquito population dynamics was considered, so the model tracked the population density of susceptible (*M*), infected (*Y*), and infectious (*Z*) mosquitoes (Figure 1). The human and mosquito infection dynamics were connected by human blood feeding of uninfected mosquitoes on infectious humans, and of infectious mosquitoes on uninfected humans. The dynamics of malaria infections in the two host populations are, thus, described by the following differential equations.

Equations for the human population:

(1)

Equations for the mosquito population:

(2)

Susceptible humans become infected when exposed to infectious mosquitoes at the rate *baZ*, where *a *is the human feeding rate and *b *is the probability of transmission [20,21]. A large proportion of infected patients (*P*) become symptomatic at the rate *ν*, an inverse of the latent period [22], these symptomatic individuals are subsequently detected and treated by passive surveillance at the clinic. Because of the delayed development of *P. falciparum *gametocytes, the rate that patients become infectious (*ξ*) after an acute attack depends on the duration of the gametocyte maturation process [7,8]. Individuals normally remain infectious for a period of time even if the infection is treated and cured. Infections with gametocytes lose their infectivity to mosquitoes at the rate *σ*, which depends in part on the different treatment regimens [13,23].

Asymptomatic asexual blood-stage infections are occasionally observed in low-endemic malaria areas [24]. Therefore, a small proportion of infected individuals (1-*P*) was assumed to become asymptomatically infected. In addition, asymptomatic cases imported from neighboring areas occur at the rate *μ*. These asymptomatic patients are likely to remain untreated and infectious for a long period, until the gametocytes are naturally eliminated at rate *r *[7]. The imported rate of asymptomatic cases was assumed to be equal to the exported rate of susceptible individuals, so that the population size remains constant.

For the mosquito population, adult mosquitoes emerge from larval habitat, which is modeled with a sinusoidal function for seasonal forcing, λ(1+sin(2πt/365). Mosquito infection occurs at rate *ac*, where *c *is the probability of transmission when they feed on infectious humans [21]. These infected mosquitoes subsequently become infectious at the rate *q*. A constant death rate, *g*, was applied to all mosquito classes.

All parameter estimates were obtained from published literature and unpublished data from the study area; except for the average mosquito birth rate, *λ*, which was estimated by fitting the model to the observed malaria occurrence data, and the waiting time to clear gametocytes under different treatment regimens, which was computed using another model (see below). Details and value estimates of parameters in the models are described in Table 2.

**Table 2:** Description of parameters and parameter estimated used in the transmission model.

Parameters	Description	Point estimates
a	Human feeding rate: Number of bites on human, per mosquito, per day, i.e., the product of the number of bites per mosquito per day and the proportion of bites on humans.	0.4 day^-1^
b	Parasite transmission probability of mosquitoes to humans: The probability that an infectious mosquito transmit the parasite to a human from a single bite.	0.6
c	Parasite transmission probability of humans to mosquitoes: The probability that a mosquito become infected from a single bite on an infectious human.	0.5
ν	The rate that an infected human becomes positive for malaria parasite: An inverse of latent period.	1/18 day^-1^
P	Treatment coverage: The proportion that infected humans receive the malaria treatment	0.99
ξ	The rate that an symptomatic human becomes infectious: An inverse of duration of gametocytogenesis	1/7 day^-1^
r	The rate that an asymptomatic human lose the infectivity: An inverse of duration of gametocyte carriage in natural infection	1/188 day^-1^
σ	The rate that an infectious human lose the infectivity with respect to treatment:	
	σ_1_: An inverse of duration of gametocyte carriage after mefloquine treatment	1/10 day^-1^
	σ_P_: An inverse of duration of gametocyte carriage after ACTs treatment	1/6 day^-1^
	σ_Q_: An inverse of duration of gametocyte carriage after primaquine follow-up treatment	1/2 day^-1^
μ	Import rate of asymptomatic cases: The proportion of imported case per person per day	0.001/365 day^-1^
g	Death rate of mosquitoes: An inverse of expected lifespan of a mosquito	1/12 day^-1^
q	The rate that an infected mosquito becomes infectious: An inverse of duration of sporogony	1/12 day^-1^
λ	Average recruitment rate of adult mosquitoes	0.33/12 day^-1^

### Model for gametocyte cycle

**Figure 2:**
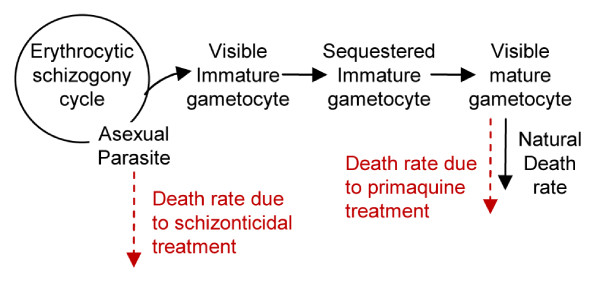
**Schematic illustration of the model for gametocytogenesis process**. An asexual parasite produces 16 new parasites at every erythrocytic schizogony cycle. A proportion of these asexual parasites convert to immature gametocytes that appear in the peripheral blood for a day before sequestrating on the blood vessel. The gametocytes become visible again when the maturation process is completed. A combination of schizonticidal and gametocidal treatment affects the gametocyte production process by eliminating the asexual parasites and the mature gametocytes.

The duration of infectivity under different drug regimens was computed using a within-host model of gametocytogenesis (Figure 2). The dynamics of the model are described by the following differential equations.

(3)

In *P. falciparum*, the erythrocytic stage takes approximately two days, at the end of which each asexual parasite, *A*_*g*_, produces approximately 16 merozoites (*θ*) [25,26]. In each cycle of erythrocytic-schizogony, all merozoites were assumed to have an equal chance of undergoing gametocytogenesis; however, only a proportion, p = 0.02, of merozoites commit to gametocytogenesis [27]. The mortality rate (*α*) is applied to the asexual parasite population due to the schizonticidal treatment. In the model, patients were assumed to receive treatment immediately after asexual parasite density reached 10^4^/μL of blood, the level that normally causes clinical symptoms among people in low-endemic areas [28-30]. After artesunate-mefloquine combination treatment, the density of asexual parasites is reduced by a factor of about 1,000 per 2-day schizogony cycle (*α*), i.e., the asexual parasites density is dropped from 10^4^/μL to 10/μL at the first cycle after treatment [31-33]. The surviving merozoites at each cycle then either convert to gametocytes or multiply into newly merozoites that continue to the next schizogony cycle. Therefore, the initial density of early stage gametocytes is a product of the conversion proportion and the net number of merozoites produced with each cycle (*pθ*). The early stage of immature gametocytes (*I*_*g*_), which are indistinguishable from asexual parasites, remain in circulation for about one day (*δ*^-1^), then sequester on blood vessels while continuing the maturation process (sequestered immature gametocytes; *S*_*g*_). It takes about 8 days (*β*^-1^) for gametocytes to mature and release to the peripheral blood (visible mature gametocytes; *G*_*g*_). The longevity of a mature gametocyte in the blood stream is approximately 3.5–4 days (*ρ*^-1^) [8].

The mortality rate of mature gametocytes due to primaquine treatment (*τ*) depends on the day of primaquine administration relative to the day of initial schizonticidal treatment (asexual parasites reach 10^4^/μL of blood). Plasma concentration of primaquine was estimated to decrease at elimination rate 0.5^3 ^per day (an 8-hour elimination half-life). Since the plasma concentration of primaquine *in vivo *is difficult to determine, primaquine was assumed to remain effective in killing 90% of mature gametocytes when the plasma concentration was above 10^-5 ^of the maximum concentration, for a net gametocyte reduction ratio of 100 per 2-day cycle, i.e., gametocyte density, which is a function of the mature gametocytes that arise from newly-emerging merozoites at each two-day cycle, eight days earlier, are reduced by 100-fold. The untreated gametocytes remaining in the blood over time were then used to determine the duration of infectiousness. Multiple realizations with different timeframes of primaquine administration were performed to determine the different durations of individual infectiousness after primaquine treatment

The duration of infectiousness is related to mature gametocyte densities and mature gametocyte longevity. The duration of infectiousness was computed based on the density of gametocytes in an individual host, using a log sigmoid relationship between gametocyte density (*G*_*g*_) and infectivity to mosquitoes [34].

(4)

**Figure 3:**
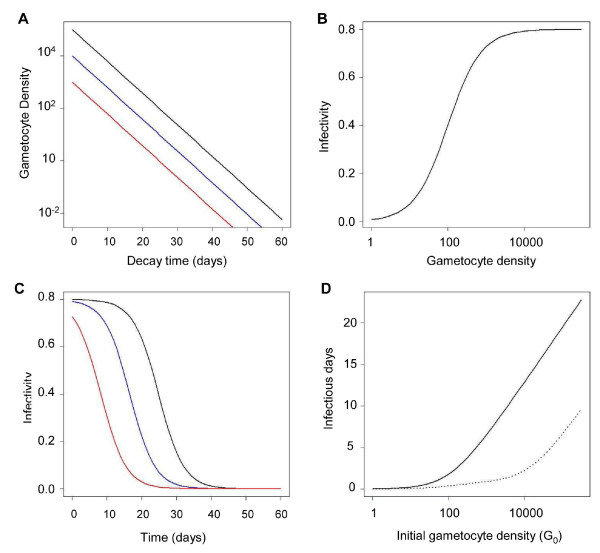
**Relationship among initial gametocyte density, gametocyte longevity, and duration of infectiousness**. (A) Changes in gametocyte density over time according to the initial gametocyte density (per μL): 10^5 ^(black line), 10^4 ^(blue line), 10^3 ^(red line). (B) Infectivity to mosquito as a function of gametocyte density (per μL). (C) Probability of infectivity to mosquito over time at different initial gametocyte densities. (D) Duration of infectiousness related to initial gametocyte density without (solid line) and with (dotted line) primaquine follow-up treatment.

With a 3.6 day half-life, gametocyte densities decline by 90% every eight days. If gametocyte densities were very high initially, then there would be virtually no drop-off in infectivity until the densities reach the S-portion of the logistic curve (Figures 3A and 3B). The time that a person remains infective (probability of infectivity more than zero) differs by about 10 days for every 10-fold difference in the initial gametocyte density (Figure 3C). Thus, for every 10-fold reduction in initial gametocyte densities achieved by curing asexual blood-stage infections alone there will be about 10-day reduction in the duration of human infectivity to mosquito. Therefore, a switch from mefloquine to artemisinin-mefloquine combination therapy (ACT) would reduce infectivity by about 10 days for every 10-fold reduction in mature gametocyte densities. Similarly, a two-day delay in appearing at the clinic could result in a 10-fold increase in gametocyte densities and a 10-day increase in infectiousness. Further reductions can only be achieved by a follow-up treatment with primaquine to reduce the densities of mature gametocytes (Figure 3D).

### Effect of optimally timing primaquine treatment at the population-level

The impact of optimally timing primaquine treatment was assessed by comparing the initial basic reproductive number for malaria derived from the population when no intervention was applied (*R*_*0*_) with the basic reproductive numbers for malaria at different scenarios of the treatment intervention (*R*_*c*_). *R*_*0 *_is an estimate of expected number of hosts infected by a single infectious person during his or her entire infectious period [35]. The magnitude of *R*_*0 *_provides insight into the transmission intensity of the disease and is often used to justify the effect of intervention programmes [36,37]. The classic formula for *R*_*0 *_of Ross and Macdonald is shown in equation 5 [35].

(5)

Where V denotes vectorial capacity, detailed definitions of other parameters are shown in Table 2.

The basic reproductive number for the control programme was calculated by modifying the Ross and Macdonald formula to consider the proportion treated with ACT (P), or with the follow-up with primaquine (Q):

(6)

Where *c *and *r *indicate the individual infectivity and duration of gametocyte carriage in natural infections, respectively. Parameters *c*_*P *_and 1/*σ*_*P *_indicate the individual infectivity and duration of gametocyte carriage, respectively, when a schizonticidal drug regimen is applied to a population; the reductions are due to the clearance of asexual parasites. The parameters *c*_*Q *_and 1/*σ*_*Q *_indicate the individual infectivity and duration of gametocyte carriage, respectively, when a primaquine follow-up regimen is applied to the population. A product of *c*_*P *_and 1/*σ*_*P*_, or *c*_*Q *_and 1/*σ*_*Q *_is the cumulative duration of infectiousness for the two different treatment regimens, which is defined as a function of gametocyte density in equation 4.

The impact of the follow-up primaquine regimen was represented by the magnitude of a ratio between *R*_*0 *_and *R*_*c *_(*R*_*0*_/*R*_*c*_). The vectorial capacity (*V*) and the transmission probability from mosquitoes to human (*b*) were assumed to be the same regardless of the intervention, so they cancel out in the ratio. Therefore, *R*_*0*_/*R*_*C *_can be calculated by the following equation.

(7)

The larger the magnitude of *R*_*0*_/*R*_*C*_, the greater the reduction in potential transmission. In addition, the relationship between the percent coverage with the primary treatment (P) and with the follow-up treatment (Q) and the ratio of *R*_*0 *_and *R*_*c *_was examined.

Finally, the optimal timing of primaquine administration was computed by finding the timing that produced the shortest infectious period in the gametocyte model. By assuming 100% coverage of follow-up primaquine treatment among symptomatic patients, the new infectious period was replaced in the initial transmission model to examine the effect of optimal timing of primaquine administration at the population-level. Also the possibility of malaria elimination in the area was determined when different elimination strategies were implemented.

## Results

### Transmission pattern

**Figure 4:**
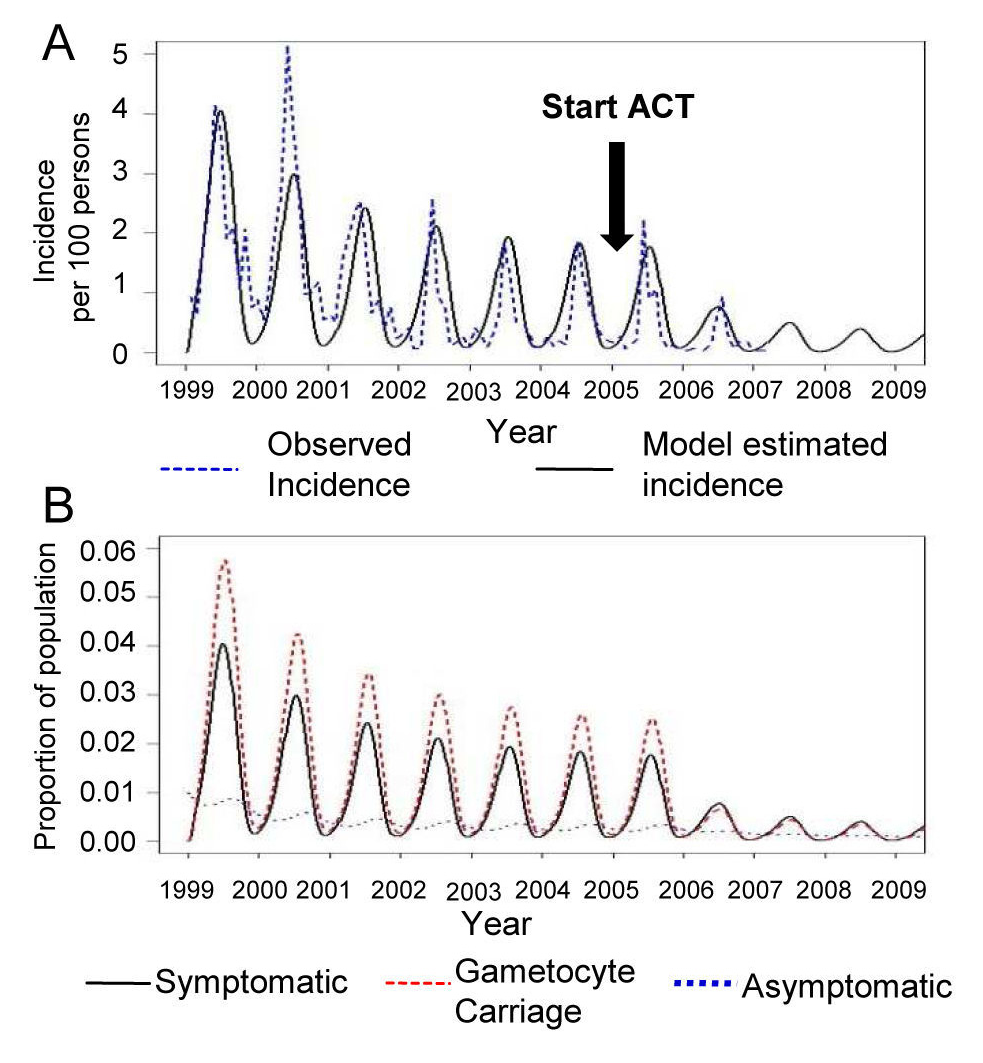
***Plasmodium falciparum *incidence and dynamic changes in proportion of the human population**. (A) Incidence of *P.falciparum *malaria in seven villages of Ratchaburi province. Solid line represents the observed incidence from 1999 to 2006. The incidence from 1999 to 2009, estimated by the transmission model, is shown by the blue dashed line. (B) Estimated changes in proportion of population in each human compartment over the 10-year period. Solid line indicates population with clinical symptoms, C_*h*_; Red dashed line represents population with gametocytaemia, G_*h*_; Blue dotted line indicates asymptomatic population, A_*h*_.

The transmission model provided a good estimate of the dynamics of malaria transmission in the study area. Over the eight-year period, the estimated incidence and observed incidence were comparable in all years except 2000, when the model significantly underestimated malaria incidence (Figure 4A). Malaria transmission in the area was strongly seasonal. Although the model assumed that 99% of the infected population received standard malaria treatment, malaria transmission only decreased gradually in the first three years before settling into a lower, stable orbit, with the annual peak incidence about 1.8 per 100 persons. The switch from mefloquine to ACT in 2005 resulted in a significant reduction in the incidence in year 2006, to less than one per 100 persons. However, estimates from the model suggest that malaria transmission will continue even after the deployment of ACT.

The changes in transmission dynamics are illustrated in Figure 4B. As is typical of low malaria transmission settings, the proportion of asymptomatic gametocyte carriers was low compared with the proportion of symptomatic individuals, which varied seasonally. While the proportion of people with gametocytaemia almost reached zero during the dry season, the model predicts that about three percent of the population remained gametocytaemic when the environment was suitable for mosquito vectors. This small proportion of gametocytaemic people could play an important role in maintaining transmission of the parasite in the area.

### Gametocyte carriage regarding primaquine treatment

**Figure 5:**
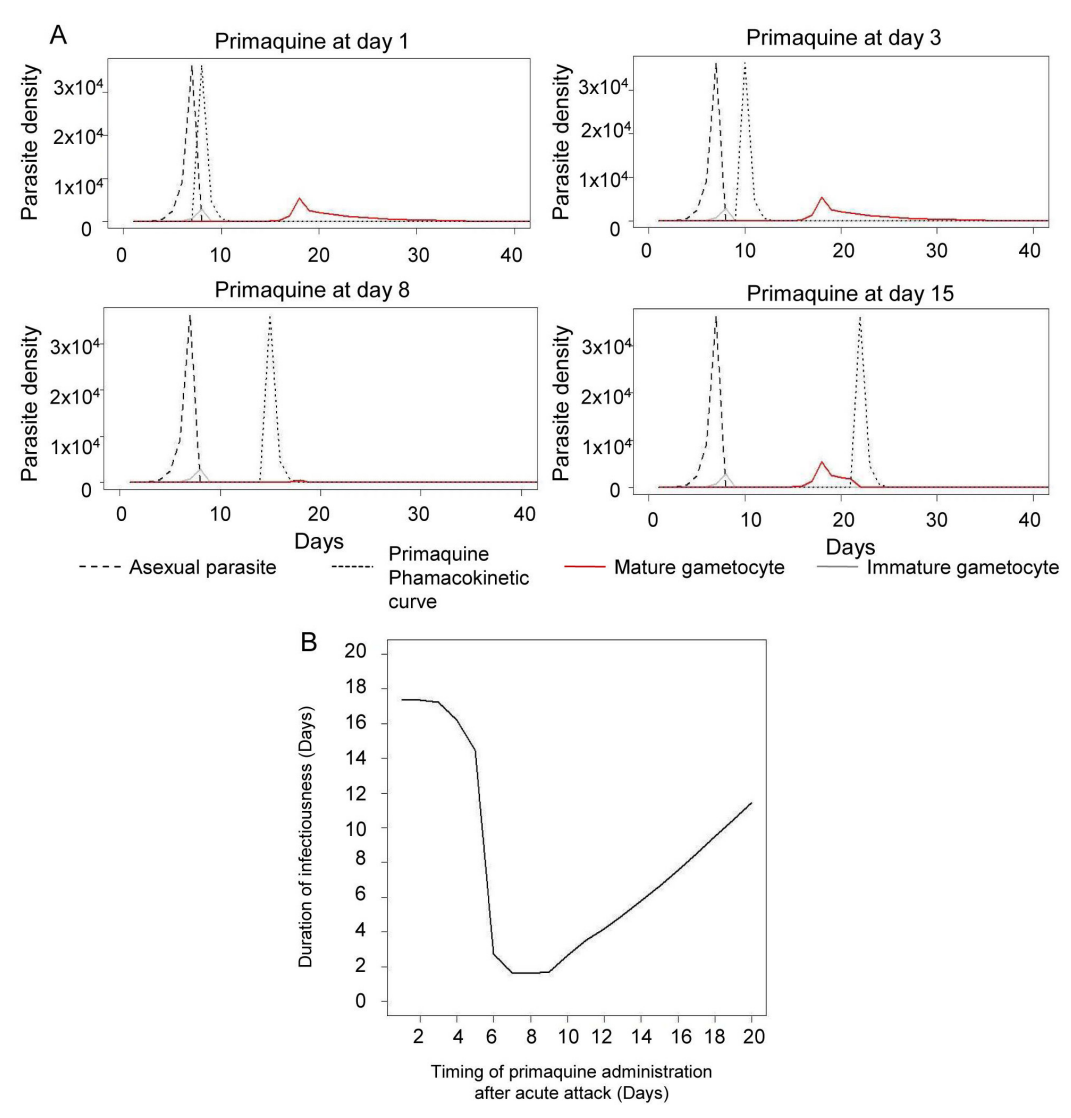
**Gametocyte dynamics and duration of infectiousness with different timings of primaquine administration**. (A) Changes in the density of each parasite developmental stage over time with different timings of primaquine administration. Pharmacokinetic curve of primaquine (dotted line) related to waves of asexual parasite, *A*_*g*_(dashed line), immature gametocytes, *I*_*g *_(grey line), and mature gametocytes, *M*_*g *_(solid red line). Initial asexual parasite (*A*_*g*_) 10/μL (B) Duration of infectivity of humans to mosquitoes over different timings of primaquine administration after acute attack.

Different malaria dynamics were observed when primaquine treatment was given at different times. Changes in the density of each parasite developmental stage over time with different primaquine treatment regimens are shown in Figure 5A. The simulated primaquine plasma concentration remained above the killing concentration for up to three days after administration. The duration and density of individual gametocytaemia differed substantially according to the timing of primaquine administration. When primaquine was given on the same day as other anti-malarial drugs, there were no mature gametocytes in the blood to be killed by primaquine, and infectiousness of the patient was not changed in comparison with no primaquine treatment. The duration of individual infectivity to mosquitoes could be as long as 14 days. In contrast, duration of infectiousness would be greatly reduced to two days if the primaquine treatment was delayed until the majority of mature gametocytes were circulating in the bloodstream (day 7 or later). On the other hand, delaying primaquine administration until day 10 or later results in an increased duration of infectiousness as mature gametocytes circulate for several days (Figure 5B).

### Optimal timing of primaquine treatment and malaria transmission

**Figure 6:**
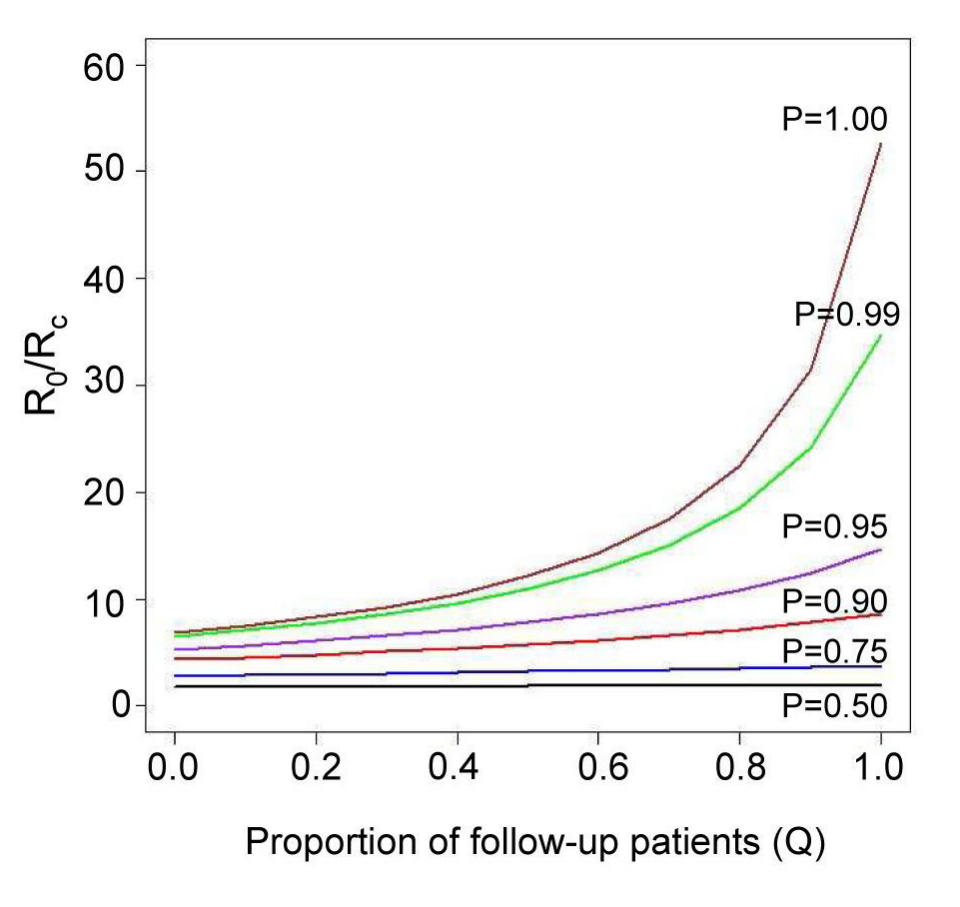
**The R_0_/Rc ratio for ACT and follow-up primaquine treatment**. The relationship between *R*_*0*_/*R*_*c *_and the proportion of *P. falciparum *patients receiving follow-up primaquine treatment (*Q*) for different proportions of patients treated with artesunate-mefloquine combination therapy (*P*).

The optimal timing of primaquine treatment showed a substantial effect in reducing malaria transmission in the area. However, the added value of the follow-up primaquine treatment was strongly correlated with the proportion of patients treated with anti-malarial drugs (P). The follow-up primaquine treatment showed a greater effect when a large fraction of clinical malaria was detected and treated. In an area with an effective detection and treatment control programme (*P *= 99%), the basic reproductive rate (*R*_*c*_) could be reduced up to 35 times by implementing the optimally timed primaquine treatment programme, compared with the initial basic reproductive rate (*R*_*0*_). More importantly, the proportional reduction in transmission that was achieved through an 8-day follow-up with primaquine in 99% of the patients was approximately equal to the proportional reductions achieved from the initial treatment. In addition, the follow-up primaquine treatment programme appeared to be effective at reducing transmission only when the coverage of the follow-up primaquine treatment was above 90% (Figure 6).

**Figure 7:**
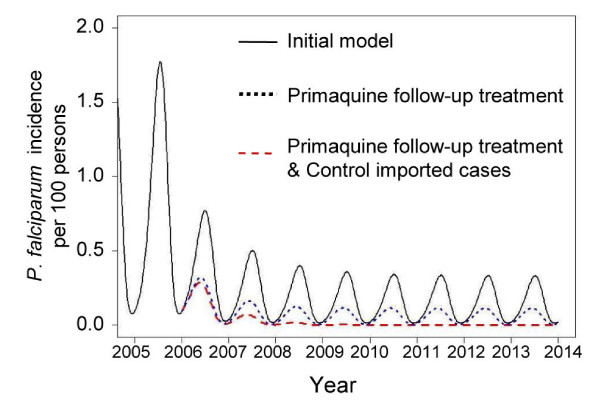
**Estimated *P. falciparum *malaria incidence for different control policies**. Solid line represents the incidence estimated by the initial model when the standard drug regimen (2-day ACTs and Primaquine at day 2) was used (solid line). The incidence significantly decreased when the timing of primaquine administration was shifted to the eighth day after initial attack (short-dashed line). The elimination of malaria transmission can be reached when the combination of both primaquine follow-up treatment and control of imported asymptomatic cases was implemented (long-dashed line).

The transmission model was re-simulated by replacing the duration of infectivity (*σ*^-1^) from 10 days to 2 days, according to the gametocyte model. The model indicated that if all individuals received primaquine at day 8 after initial acute attack, the malaria incidence in the area would be reduced substantially (Figure 7). However, changing the primaquine administration regimen alone may not be enough to eliminate *P. falciparum *malaria in the area; the incidence of malaria still persisted during the wet season, largely because of imported malaria. By including an intervention focused on the effective detection and treatment of imported asymptomatic infections, the incidence of malaria in the area could reach zero within three years after the combination programme was introduced (Figure 7).

## Discussion

Mathematical models were constructed to understand the transmission dynamics of *P. falciparum *malaria in an area where access to health-care has significantly reduced malaria transmission. Active malariometric surveys suggest that a very high fraction of clinical episodes in the area, perhaps higher than 97% are promptly treated [38]. Prompt and effective treatment may be a very cost-effective strategy for malaria control in low or unstable malaria transmission settings, because most individuals are likely to develop acute febrile illness after *P. falciparum *infection [39-41]. Significant reductions in mortality and morbidity of malaria after deploying a strategy of early detection and prompt treatment has been documented [42]. However, model simulations find that, despite significant reductions in incidence, *P. falciparum *transmission is likely to continue. This suggests that the elimination of malaria may be difficult even in an area where malaria health systems are highly effective.

Given the fact that multiple surveys of large swaths of the population are unable to find individuals infected asymptomatically, gametocyte carriage in the absence of asexual parasites seems to be vital for maintaining transmission in this heavily controlled area [7,24]. Findings of our model suggest that there is a strong seasonal fluctuation in the population of residual gametocyte carriers, which is consistent with previous observations in other low transmission areas [7,9]. An interesting finding is that while the prevalence of gametocytes is relatively small during the dry season, it does not drop to zero. Presumably then these cases are responsible for the source of mosquito infection at the start of wet season [7,9].

Gametocyte reduction is of great interest for malaria control, particularly in low-endemic malaria areas. One of the strategies for the malaria elimination programme, recommended by the WHO, is to identify and treat all malaria patients as well as to reduce onward transmission caused by gametocytaemia [6]. The policy change from mefloquine treatment to artesunate-mefloquine has been shown to reduce transmission. Artemisinin combination therapies can reduce the asexual parasite burden 100 times faster than mefloquine, which can subsequently inhibit development of more mature gametocytes [31-33,41]. The gametocyte model indicates that there is still an added value of the follow-up primaquine treatment even when the initial gametocyte density is small due to the switch from mefloquine to ACTs. In addition, in areas where artemisinin combination therapy has been used, gametocyte carriage is still common in the 7–21 days following treatment [13,30,41,43-45]. In many countries, a single oral dose of primaquine is included in the standard anti-malarial drug regimen with the aim of further reducing gametocyte carriage, even when artemisinin-based therapy is used [6,12].

However, while in these areas primaquine can be extremely effective at clearing gametocytes that persist after treatment with schizonticidal agents [13,14,43], the timing and duration of gametocyte carriage and subsequent infectiousness have not been considered carefully when primaquine is deployed as a transmission-blocking agent. Findings of the gametocyte model indicate that the effectiveness of primaquine in reducing the duration of infectiousness depends critically on timing. Primaquine is most beneficial when the administration is delayed, about eight days following initial treatment, to coincide with the release of a large cohort of mature gametocytes into the blood, which emerged from a large number of merozoites during an acute attack. The effect of primaquine is significantly reduced when the drug is given too early or too late. Although an immediate primaquine treatment can affect a small cohort of mature gametocytes that emerge from the first crop of merozoites that appear in circulation at the time of an acute attack, primaquine will be cleared from the system before the largest cohort of gametocytes mature. If primaquine is given too late, mature gametocytes will be able to circulate and infect mosquitoes until the drug is administered.

The benefits of optimally timed primaquine are greatest in those areas where the early treatment programme to cure asexual blood stage infections is very successful; a high fraction of clinical malaria episodes are expected to receive the standard treatment within one to three days after acute attack. In such areas, optimally timing primaquine administration shows a potential impact on overall malaria transmission at the population-level. The current results show that follow-up primaquine treatment can reduce the duration of infectiousness over the existing strategy of using artesunate-mefloquine alone, with a combined total net reduction in transmission of 98%, a 95-fold reduction in *R*_*0*_. An important observation is that the added value of optimally timed primaquine can have relatively large effects on reducing transmission only if a high fraction of patient infections are treated and cured with first-line anti-malarial drugs (i.e. when *P *is high), suggesting that the first emphasis should be on treating those with clinical malaria. Because primaquine effectively reduces transmission only in those patients who have cleared their asexual parasites, and because the average duration of an asymptomatic infection is approximately six months, the benefit of reducing the duration of gametocyte carriage is of little importance unless at least 90% of clinical malaria episodes are effectively treated. Treatment to clear asexual parasites and prevent asymptomatic infections can only reduce the duration of infectiousness insofar as the gametocytes are also cleared. In such situations, primaquine can be very effective at further reducing the duration of infectiousness, and the added value of good follow-up with primaquine treatment has nearly the same proportional effects on potential transmission as does the primary treatment.

In addition, findings from the model indicate that when all symptomatic *P. falciparum *patients receive the follow-up primaquine treatment at day eight, the *P. falciparum *malaria incidence can be reduced nearly to zero. However, the model suggests that a small number of undetected imported cases can pose a big threat for malaria elimination. To reach the elimination goal, vigilance to detect and cure imported asymptomatic cases may also be required. These findings support the WHO recommendations for a malaria elimination programme [6].

Mathematical modeling has been widely used to model transmission dynamics of malaria, and control interventions [46]. However, findings from the models require a careful interpretation. This study intends to construct simple models that provide a valuable insight into the feasibility of malaria elimination in a low malaria transmission area. Results from the models should be considered as approximations that are likely to differ because of the natural variability under field conditions.

In addition, although the model assumed that the level of individual infectiousness follows the log-sigmoid relationship with the gametocyte density among non-immune adults, the duration of infectiousness in the analysis may be underestimated. Infectivity to mosquitoes is observed even when gametocyte densities fall below detection level by microscopy or by a molecular method [24,47]. However, the trend of duration of infectiousness over different timings of primaquine administration does not change when different infectivity levels are applied. The model also does not take into account the additional gametocyte carriage from recrudescent infections. Though gametocytaemia is estimated to be greater in recrudescent infections than in primary infections, in an area where artesunate-mefloquine combination therapy is used, few recrudescent infections are expected [33,48]. Lastly, the model assumes that most infected individuals develop clinical symptoms and are treated. In low and unstable transmission areas this assumption is generally correct; however pockets of higher transmission may exist, and the importance of asymptomatic asexual blood-stage infections in these areas in continuing transmission over the dry season can be significant. While surveys suggest that there are basically no asymptomatic carriers in the region in question, studies in other areas have found that sub-patent infections can persist for many months [9,10,49]. Thus, while the models suggest that optimally-timed primaquine administration can significantly impact the incidence of malaria in a low-transmission area well served by health centers, asymptomatic individuals in the area and not just imported carriers may also play a significant role in sustaining transmission and should be considered in any elimination plan.

## Conclusion

Mathematical models constructed in this study pose an important and testable hypothesis regarding existing control programmes in areas with good health systems where malaria transmission persists. The transmission-blocking effect of primaquine and the timing of its administration should be carefully scrutinized. Given the risks associated with primaquine, it may not be worth giving, except in areas where the early detection and prompt treatment programme is highly effective. In such areas, primaquine should be administered at an appropriate time, or a long acting 8-aminoquinoline should be considered, and combined with surveillance to catch imported malaria it could lead to local malaria elimination. Randomized controlled trials are recommended to determine the most-effective timing of primaquine administration in order to decrease malaria transmission, which is important for planning malaria elimination programmes in low malaria transmission areas.

## Competing interests

The authors declare that they have no competing interests.

## Authors' contributions

SL and DLS conceived and designed the experiment. SL analysed the data. PS, SY, NT, and WM managed the data set. SL, DLS, EK, LLH, and JHM wrote the paper. All authors read and approved the final manuscript.
